# Sublingual AKBA Exerts Antidepressant Effects in the Aβ-Treated Mouse Model

**DOI:** 10.3390/biom11050686

**Published:** 2021-05-03

**Authors:** Maria Grazia Morgese, Maria Bove, Matteo Francavilla, Stefania Schiavone, Stefania Dimonte, Anna Laura Colia, Matteo Bevilacqua, Luigia Trabace, Paolo Tucci

**Affiliations:** 1 Department of Clinical and Experimental Medicine, University of Foggia, 71122 Foggia, Italy; maria.bove@unifg.it (M.B.); stefania.schiavone@unifg.it (S.S.); stefania.dimonte@unifg.it (S.D.); annalaura.colia@unifg.it (A.L.C.); luigia.trabace@unifg.it (L.T.); paolo.tucci@unifg.it (P.T.); 2 STAR*Facility Centre, Department of Agriculture, Foods, Natural Resources and Engineering, University of Foggia, 71122 Foggia, Italy; matteo.francavilla@unifg.it; 3 Pharmaceutica San Marco, Via Vittor Pisani, 6 20142 Milan, Italy; matteo.bevilacqua38@gmail.com

**Keywords:** 3-O-acetyl-11-keto-β-boswellic acid, beta amyloid, depressive-like behavior, kynurenine, glutamate, sublingual route of administration

## Abstract

The 3-O-acetyl-11-keto-β-boswellic acid (AKBA) is the most active compound of *Boswellia serrata* proposed for treating neurodegenerative disorders, including Alzheimer’s disease (AD), characterized in its early phase by alteration in mood. Accordingly, we have previously demonstrated that an intracerebroventricular injection of soluble amyloid beta _1-42_ (Aβ) peptide evokes a depressive-like phenotype in rats. We tested the protective effects of AKBA in the mouse model of an Aβ-induced depressive-like phenotype. We evaluated the depressive-like behavior by using the tail suspension test (TST) and the splash test (ST). Behavioral analyses were accompanied by neurochemical quantifications, such as glutamate (GLU), kynurenine (KYN) and monoamines, and by biochemical measurements, such as glial fibrillary acid protein (GFAP), CD11b and nuclear factor kappa B (NF-kB), in mice prefrontal cortex (PFC) and hippocampus (HIPP). AKBA prevented the depressive-like behaviors induced by Aβ administration, since we recorded a reduction in latency to initiate self-care and total time spent to perform self-care in the ST and reduced time of immobility in the TST. Likewise, the increase in GLU and KYN levels in PFC and HIPP induced by the peptide injection were reverted by AKBA administration, as well as the displayed increase in levels of GFAP and NF-kB in both PFC and HIPP, but not in CD11b. Therefore, AKBA might represent a food supplement suitable as an adjuvant for therapy of depression in early-stage AD.

## 1. Introduction

The 3-O-acetyl-11-keto-β-boswellic acid (AKBA) is the most active triterpenoid compound from extracts of Boswellia serrata. This compound can be extracted from incense, or frankincense or olibanum, a resin produced by Boswellia plants, of the Burseraceae family, known since ancient times for its healing properties. The use of Boswellia has been proposed for several inflammatory conditions, such as a rheumatoid arthritis, osteoarthritis, chronic colitis, ulcerative colitis, Crohn’s disease and bronchial asthma [1]. In addition, the genus Boswellia, comprising about 20 species, has been studied as a new candidate for neurodegenerative disorders, including Alzheimer’s disease (AD) [2]. In keeping with this hypothesis, it has been reported that AKBA holds antioxidant and anti-inflammatory properties and recently it has been tested as a disease-modifying therapeutic potential drug for AD by using a transgenic animal model [3]. In this light, it has been shown that this compound was able to reverse cognitive impairments and to decrease cerebral amyloid beta (Aβ) production and plaque burden in APPswe/PS1dE9, an AD genetic model. In addition, AKBA seems to have anti-amyloidogenic action [2] and has also been shown to inhibit the deposition of Aβ and tau proteins [4], and by reducing Aβ levels, it has been reported to be effective in ameliorating cognitive impairment in the rodent model [3]. However, AD is a complex neurodegenerative disease also characterized in its early phase by alteration in mood. In particular, it has been shown that a depressive state can largely precede cognitive decline [5,6] and such an event can be associated with increased levels of Aβ soluble neurotoxic species [7]. In this regard, we have previously shown that an intracerebroventricular (icv) injection of soluble Aβ_1-42_ generates a depressive like-phenotype in rats that is featured by depressive-like behavior accompanied by altered monoamine content in prefrontal-cortical (PFC) and hippocampal (HIPP) areas, increased glial activation and neuroinflammation [8,9,10]. Indeed, many lines of evidence suggest that some types of depression are associated with an increased inflammation state [11,12]. Furthermore, a depressive state has been evoked in rodents by using toxins able to generate a robust inflammatory response [13,14]. Recent studies have shown that AKBA ameliorates cerebral injury outcomes and counteracts cerebral damages associated with neuroinflammation in rodent models [15]. In keeping with these reports, the neuroprotective property of AKBA has drawn increasing interest in recent years. Therefore, in our study we decided to test the protective effects of AKBA by using the mouse model of an Aβ-induced depressive-like phenotype. In this animal model, we evaluated the depressive-like behavior by using two validated behaviours in mice, such as the tail suspension test (TST) and the splash test (ST). Furthermore, along with neuroinflammation, AD is also characterized by excitotoxic levels of extracellular glutamate (GLU) [16,17]. Likewise, alterations in glutamatergic function have also been postulated as an alternative to the monoaminergic hypothesis of depression [18]. Thus, in the present study, we evaluated the cortical and hippocampal levels of GLU in Aβ-treated mice and we later tested the ability of AKBA to modulate this neurochemical parameter. On the other hand, we have previously found that the icv injection of the peptide was accompanied by increased levels of kynurenine (KYN). This molecule is produced from tryptophan after enzymatic bioconversion operated by indoleamine 2, 3-dioxygenase (IDO) enzymes. The metabolic shift from tryptophan metabolism towards KYN and its derivatives, such as kynurenic or quinolinic acids, instead of towards 5-HT has been proposed as another possible biological mechanism under evaluation for explaining a depressive state [19]. Interestingly, a crucial crosstalk between the KYN pathway and glutamatergic function has been described [20]. Thus, the role played by AKBA in regulating the interconnection between those biological substrates has been investigated in the Aβ-treated animal model. Nonetheless, glial cells play a crucial role in maintaining the homeostasis of GLU, along with the regulation of pro-inflammatory biomarkers production after toxic brain insults, such as extra-physiological Aβ levels. Indeed, astrogliosis and microglial activation have been described in vivo after icv of Aβ by our group and other researchers [9]. Thus, to understand the putative neuroprotective mechanism of action of AKBA, we quantified biological biomarkers associated with glial activation such as glial fibrillary acidic protein (GFAP), for astrocytes, and CD11b, a marker of activated macrophages and microglia. Ultimately, it has been shown that AKBA might exert its beneficial effects by suppressing nuclear factor kappa-B (NF-kB) and NF-kappa B-regulated gene expression [21]. NF-kB is a heterodimeric transcription factor playing a crucial role in orchestrating immune response and neuroinflammation. This transcription factor is activated by prostanoids and pro-inflammatory cytokines that consequently activate NF-kB generating a vicious cycle [22]. Therefore, the role played by NF-kB in inducing the Aβ-depressive phenotype and in turn the effects of AKBA on these mechanisms have been investigated.

## 2. Materials and Methods

### 2.1. Animals

The experiments were carried out by using one group of C57/Bl6 male mice of 8–10 weeks old and another group of C57/Bl6 male mice of 10–12 weeks old (Envigo, San Pietro al Natisone, Italy). They were housed at constant room temperature (22 ± 1 °C) and relative humidity (55 ± 5%), under a 12h light/dark cycle. Water and food were available ad libitum. Procedures involving animals and their care were conducted in conformity with the institutional guidelines of the Italian Ministry of Health (D.L. 26/2014), the Guide for the Care and Use of Laboratory Animals: Eighth Edition, the Guide for the Care and Use of Mammals in Neuroscience and Behavioral Research (National Research Council, 2004), the Directive 2010/63/EU of the European Parliament and of the Council of 22 September 2010 on the protection of animals used for scientific purposes, in accordance with ARRIVE guidelines. Animal welfare was daily monitored through the experimental period and all efforts were made to minimize the number of mice used and their suffering. The experimental protocol was approved by the Italian Ministry of Health (approval number 665/2019-PR, protocol n. B2EF8.23).

### 2.2. Surgery and Amyloid Beta Administration

The Aβ 1-42, obtained from Tocris (Bristol, UK), was dissolved in sterile double-distilled pyrogenic-free water, as vehicle, to obtain a final concentration 4 µM [8]. Mice were anesthetized with a solution (0.85 in mL/kg, i.p.), containing ketamine (Sigma Aldrich, Milan, Italy, 100 mg/10 mL), xylazine (Sigma Aldrich, Milan, Italy, 100 mg/10 mL) and acepromazine (prequillant, A.T.I. Azienda Terapeutica Veterinaria S.r.l., 10 mg/10 mL) dissolved in saline. Animals were secured in a stereotaxic frame (David Kopf Instruments, Tujunga, CA, USA) and the peptide injection was accomplished in the lateral ventricle of mice at the following coordinates: AP = −0.2, ML = +1 and DV = −2 relative to bregma, according to the atlas of Paxinos and Franklin [23]. The icv infusion was carried out by using a 25 µL Hamilton microsyringe connected to the infusion pump at constant flow rate of 2 µL/min for 1.30 min (volume injected 3 µL). The needle was left in place for other 3 min to avoid reflux. The control group (SHAM) received only vehicle, considering that according to our previous observations effects retrieved by the injection of reverse Aβ 42-1 were similar to the vehicle alone. At the time of dissection, the correct needle track was assessed. All in vivo and ex vivo experimental procedures were conducted in mice 7 days after surgery.

### 2.3. AKBA Administration and Central Bioavailability Study

AKBA (Cayman Chemical Company) was dissolved in sunflower oil, this vehicle was chosen in order to maintain a better tasting and higher density of the final solution thus reducing reflux through the digestive tract. The dose of 5 mg/kg was chosen based on preliminary experiments indicating that this dose was the lowest showing antidepressant effects on intact mice (unpublished observations) and on other previously published data on animal models [24]. Ten µL of the solution (or vehicle alone) was sublingually administered to mice. For intranasal administration, requiring more hydrophilic solution, AKBA was dissolved in DMSO and saline (1:3, *v*/*v*), and given to mice at the same final dose of 5 mg/kg. Cerebral AKBA was quantified in a separate subset of mice at 5, 15 and 30 min after its administration, either sublingually or intranasally.

All behavioral experiments were conducted at 5 or 30 min after sublingual administrations of AKBA or vehicle in either SHAM or Aβ-treated mice. Based upon behavioral outcomes observed, neurochemical and biochemical quantifications were performed 30 min after AKBA or vehicle administration in either SHAM or Aβ-treated mice.

#### AKBA Quantification by GC-MS/IT

After administration, whole brains were put in 1 mL of a solution of chloroform/methanol (1:1 *v*/*v*), sonicated and then homogenates were centrifuged at 4 °C for 20 min (10,000 rpm). The pellets were then removed, and the remaining supernatants were dried on sodium sulphate anhydrous, filtered with 0.20 µm PTFE syringe filters and used for chemical analysis. The quantification of 3-O-acetyl-11-keto-β-boswellic acid (AKBA) was performed by using GC-MS/IT equipment composed of a gas chromatograph GC-7890B (Agilent Technologies, Santa Clara, CA, USA) coupled with an ion trap mass spectrometer IT-240 (Agilent Technologies).

The gas chromatograph was equipped with a VF-5 ms (Agilent J&W GC Columns) capillary column, 30 m × 0.25 mm i.d. × 0.25 µm. The column oven was set at 50 °C for 1 min after injection; then the temperature was increased from 50 °C to 300 °C with a ramp of 5 °C/min and a hold time of 10 min. The purge process was performed at the end of each run, increasing the oven temperature from 300 °C to 325 °C at 10 °C/min and a hold time of 1.5 min. The total time for each run was approximately 65 min. The injection volume was 2 µL (splitless) with a continuous flow rate of 1 mL/min of ultra-high purity helium (BIP^®^) and an injector temperature of 250 °C.

**Figure 1:**
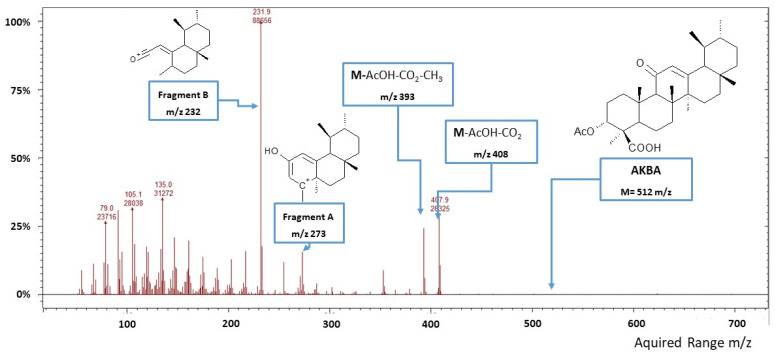
Mass spectrum (full scan mode) of 3-O-acetyl-11-keto-β-boswellic acid (AKBA).

The MS electron multiplier voltage was set at 1450 V, and an ionization time of 65,000 µs was used, running in the electron impact (EI) mode. The transfer line, ion trap and manifold temperatures were set at 270 °C, 180 °C and 70 °C, respectively. The mass spectrometer firstly operated in full scan mode (50–700 *m*/*z*) with the ionizing voltage at 70 eV. The mass spectrum of AKBA standard (RT = 53.53 min, MW = 512 g/mol) showed characteristic mass fragments at *m*/*z* 408 (30%), 393 (28%), 273 (22%) and 232 (base peak) (Figure 1). Peaks at *m*/*z* 408 and *m*/*z* 393 were identified as M-AcOH-CO2 and M-AcOH-CO2-CH3. The peak at *m*/*z* 273 was identified as fragment A, resulting from retro-Diels–Alder fragmentation, while the peak at *m*/*z* 232 was identified as fragment B, generated from McLafferty fragmentation. These characteristic fragments were selected as a mass target to develop the MS acquisition method operating in single ion scan mode (SIS) to improve the LOD and LOQ for AKBA quantification. A calibration curve was made by linear interpolation of standards prepared by diluting AKBA in CH2Cl2 at seven concentration levels (0.5, 5, 50, 100, 250, 500 and 1000 ng/mL). Mass data were acquired and processed using the MS-Workstation software version 7.0.1 (Agilent Technologies). All analyses were performed in triplicate.

### 2.4. Behavioural Tests

#### 2.4.1. Open Field Test

Mice were placed in an open field arena and allowed to explore for 30 min [25]. After each trial, the arena floor was cleaned with 70% ethanol to avoid inter-assay bias. The movements of mice were video recorded and analyzed by ANY-maze tracking software (Ugo Basile-Varese, Gemonio, Italy). Locomotion was evaluated through total crossing measurements.

#### 2.4.2. Splash Test

The splash test was carried out as previously reported [25]. A 10% sucrose solution was sprayed on the dorsal coat of the animal positioned alone in a Plexiglas cage (30 × 16 × 19 cm). The viscosity of the sucrose solution elicited robust self-grooming considered a self-care behavior. The test was videotaped and later an observer, blind to the trial, scored for latency to the first grooming event and duration of self-care behavior during the period of the test (5 min).

#### 2.4.3. Tail Suspension Test

The test was performed as previous reported [26]. Briefly, mice were left in the testing room 1 h prior to performing the test in order to allow for acclimation. Then animals were suspended by attaching their tails with adhesive tape (approximately 1 cm from the tip of the tail) to a suspension bar. The test was video recorded for 6 min, while immobility time was measured for the last 5 min. Mice were considered to be performing immobility when passively hanging motionless.

### 2.5. Post-Mortem Tissue Analysis

Anesthetized animals were sacrificed by cervical dislocation. Brains were immediately removed and kept on ice for dissection of PFC and HIPP, according to the atlas of Paxinos and Franklin. Tissues were frozen and stored at −80 °C until analyses were carried out. Samples (PFC and HIPP) were homogenized (1:10 *w*/*v*) at 4 °C using a PBS buffer containing a 1:100 protease and phosphatase inhibitor cocktail (HALT inhibitors, Thermo Fisher Scientific, Cleveland, OH, USA) for biochemical analyses or perchloric acid 0.1 M for neurochemical analyses. Homogenates were centrifuged at 13,000× *g* at 4 °C for 20 min, and supernatants were used for further determinations.

#### 2.5.1. Neurochemical Quantifications

5-HT, noradrenaline (NA), and KYN levels were measured in the PFC and HIPP of mice by HPLC coupled with an electrochemical detector (Ultimate ECD, Thermo Scientific Dionex, Milan, Italy) as already reported [27,28]. Separation was performed by a LC18 reverse phase column (Kinetex, 150 mm × 3.0 mm, ODS 5 μm; Phenomenex, Castel Maggiore-Bologna, Italy). The detection was accomplished by a thin layer amperometric cell (Thermo Scientific Dionex, Milan, Italy) with a 5 mm diameter glassy carbon electrode at a working potential of 400 mV (5-HT and NA) or 0.750 mV (KYN) vs. Pd. The mobile phase consisted of an aqueous buffer containing 75 mM NaH2PO4, 1.7 mM octane sulfonic acid, 0.3 mM EDTA, and acetonitrile 10%, buffered at pH 3.0. The flow rate was kept at 0.7 mL·min-1 by an isocratic pump (Shimadzu LC-10 AD, Kyoto, Japan). Data acquisition and integration were performed by using Chromeleon software (version 6.80, Thermo Scientific Dionex, Milan, Italy) [29]. GLU concentrations were determined by HPLC coupled with fluorescence detection (emission length 460 nm; excitation length 340 nm), as previously published [30]. Analyses were carried out using an LC18 reverse phase column (Kinetex, 150 mm × 3.0 mm, ODS 5 μm; Phenomenex, Castel Maggiore, Bologna, Italy) and detection was accomplished by pre-column derivatization with ophthalaldehyde/mercaptopropionic acid. The mobile phase consisted of a 50 mM sodium acetate buffer, at pH 6.95, with gradient methanol increasing linearly from 2 to 30% (*v*/*v*) over a 40 min run. The gradient flow rate was maintained by a pump (JASCO, Tokyo, Japan) at 0.5 mL/min. Results were analyzed by Borwin software (version 1.50; Jasco, Cremella, Italy) and the amino acid concentration was expressed as µM. All data were normalized for total area weight and were expressed as concentration/mg of tissue.

#### 2.5.2. Western Blotting Quantification

The total amount of proteins was measured in homogenates by using Pierce BCA Assay (Thermo Fisher Scientific, Cleveland, OH, USA). Forty µg of the total lysate protein were separated by SDS-PAGE precast gels (Bio-Rad Laboratories Inc., Segrate (MI), Italy), transferred onto nitrocellulose membranes (Bio-Rad Laboratories Inc, Segrate (MI), Italy) and then blocked for 1 h in blocking buffer (SigmaAldrich, Milan, Italy) [31]. Rabbit polyclonal antibody against GFAP (Dako Products, USA; 1:2000), rabbit monoclonal antibody against CD11b (Abcam, Cambridge, MA, USA; ab133357, 1:1000) and mouse monoclonal antibody against NF-kB p65 (Santa Cruz Biotechnology, Dallas, Texas, USA; 1:2000) were used to incubate the membranes overnight at 4 °C. After HRP-conjugated specific antibody incubation, ECL reagent (Bio-Rad Laboratories Inc., Segrate (MI), Italy) was added to the immune complex and chemiluminescence was detected by ChemiDoc MP system (Bio-Rad Laboratories Inc., Segrate (MI), Italy). Optical densities of the bands were measured using ImageJ software (http://rsb.info.nih.gov/ij/ accessed on 15 March 2021) and normalized against bands relative to β-actin (1:5000, Abcam, Cambridge, UK).

### 2.6. Statistical Analyses

Data were expressed as mean ± SEM. Experiments were analyzed using two-way (bioavailability data) or one-way analysis of variance (ANOVA) followed by Tukey’s multiple comparisons test. AUCs data were analyzed by the unpaired Student’s *t*-test. All analyses were performed by using GraphPad Prism 5 (GraphPad Software, San Diego, CA, USA). Differences among groups were considered significant at values of *p* < 0.05.

## 3. Results

### 3.1. Evaluation of AKBA in Brain after Sublingual or Intranasal Administration

**Figure 2:**
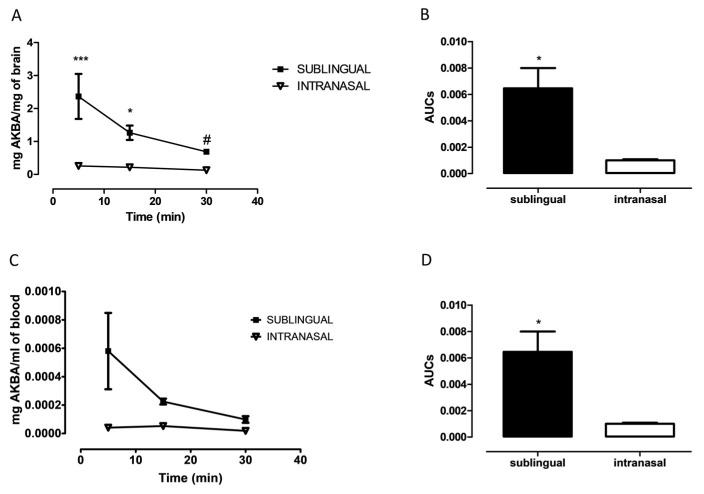
Cerebral AKBA quantification after sublingual or intranasal administration. (**A**) Cerebral AKBA levels (mg AKBA/mg of brain) at 5, 15, and 30 min after sublingual (black square, n = 4, 15 n = 4, 30 n = 5), or intranasal (reverse empty triangle, 5 n = 5, 15 n = 5, 30 n = 5) administration. (**B**) Cerebral AUCs of sublingual (black bar, n = 5), and intranasal (empty bar, n = 5) administration. (**C**) Plasmatic AKBA levels (mg AKBA/mg of brain) at 5, 15, and 30 min after sublingual (black square, n = 4, 15 n = 4, 30 n = 5), or intranasal (reverse empty triangle, 5 n = 5, 15 n = 5, 30 n = 5) administration. (**D**) Plasmatic AUCs of sublingual (black bar, n = 5), and intranasal (empty bar, n = 5) administration. Two-way ANOVA followed by Bonferroni multiple comparisons test * *p* < 0.05 sublingual vs. intranasal at 5 min; *** *p* < 0.001 sublingual vs. intranasal. One-way ANOVA followed by Tukey’s multiple comparisons test, # *p* < 0.05 5 vs. 30 min for sublingual.

In order to evaluate if AKBA was able to cross the blood brain barrier we quantified cerebral and plasmatic AKBA levels at 5, 15, and 30 min after its sublingual or intranasal administration. As shown in Figure 2A, AKBA levels in the whole brain of treated mice was significantly higher after sublingual administration compared to intranasal, as revealed by two-way ANOVA using concentrations and the route of administration as variables (Figure 2A, two-way ANOVA, F_1,22_ = 36.76 followed by Bonferroni post hoc test 5 min *p* < 0.001 and 15 min *p* < 0.05 sublingual vs. intranasal). Furthermore, the highest concentrations were detected at 5 min after administration. However, such an endpoint was significantly different from the 30 min one only for sublingual administration (one-way ANOVA, F_2,10_ = 5.061, followed by Tukey’s multiple comparison test *p* < 0.05, sublingual: 5 vs. 30 min). Regarding Figure 2B, when AUCs were compared, the Student’s *t*-test revealed a significant difference between groups (Figure 2B, unpaired Student’s *t*-test, *p* < 0.001, sublingual vs. intranasal). Parallel evaluation was performed for plasmatic AKBA levels. As shown in Figure 2C, AKBA levels in blood, considering concentrations and the route of administration as variables, were significantly higher after sublingual administration compared to intranasal (Figure 2C, two-way ANOVA, F_1,12_ = 8.564 followed by Bonferroni post hoc test 5 min *p* < 0.05 sublingual vs. intranasal). Likewise, AUCs evaluation indicated that this value was higher after sublingual administration of AKBA to mice (Figure 2D, unpaired Student’s t-test, *p* < 0.05, sublingual vs. intranasal).

### 3.2. Antidepressant-Like Effect of Sublingual AKBA in Aβ-Treated Mice

**Figure 3:**
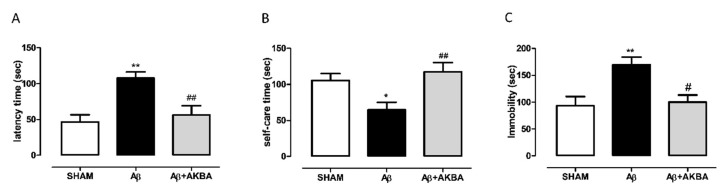
Behavioral effects of AKBA administration in Aβ-treated mice by using the splash test and the tail suspension test. (**A**) Latency to leak (sec) in the splash test of mice 7 days after icv injection of vehicle (SHAM, 5 µL, white bar), Aβ (Aβ, 4 µM, black bar), and Aβ+AKBA (Aβ, 4 µM + sublingual AKBA, 5 mg/kg, gray bar), (SHAM n = 9, Aβ n = 9, Aβ+AKBA n = 10). (**B**) Time spent performing self-care (sec) in the splash test of mice 7 days after icv injection of vehicle (SHAM, 5 µL, white bar), Aβ (Aβ, 4 µM, black bar), and Aβ+AKBA (Aβ, 4 µM + sublingual AKBA, 5 mg/kg, gray bar), (SHAM n = 9, Aβ n = 9, Aβ+AKBA n = 10). (**C**) Immobility time (sec) in the tail suspension test of mice 7 days after icv injection of vehicle (SHAM, 5 µL, white bar), Aβ (Aβ, 4 µM, black bar), and Aβ+AKBA (Aβ, 4 µM + sublingual AKBA, 5 mg/kg, gray bar), (SHAM n = 12, Aβ n = 9, Aβ+AKBA n = 6). One-way ANOVA followed by Tukey’s multiple comparisons test, ** *p* < 0.01, * *p* < 0.05 Aβ versus SHAM, and ## *p* < 0.01, # *p* < 0.05 Aβ+AKBA versus Aβ.

We have previously shown that an icv injection of Aβ peptide is able to evoke a depressive-like phenotype in rats. Here, we tested to see if the icv injection could induce a depressive-like behavior in mice. As shown in Figure 3A, the peptide injection induced a significant increase in latency to leak in the splash test, while when mice received AKBA 30 min before testing such an outcome was reverted (one-way ANOVA F_2,25_ = 9.049 followed by Tukey’s multiple comparison test *p* < 0.01 SHAM vs. Aβ, and Aβ vs. Aβ+AKBA). In addition, the peptide evoked a reduction in time spent performing self-care, and AKBA administration prevented such an effect (Figure 3B one-way ANOVA F_2,25_ = 6.128 followed by Tukey’s multiple comparison test *p* < 0.05 SHAM vs. Aβ, and *p* < 0.01 Aβ vs. Aβ+AKBA). However, when behavioral outcomes were evaluated at 5 min after AKBA treatment, the depressive-like behavior induced by Aβ administration was not reverted (Supplementary Material, Figure S1). In order to confirm the data obtained through the splash test, the tail suspension test was carried out. As shown in Figure 3C, the peptide induced a significant increase in the immobility time compared to SHAM mice, and AKBA administration prevented such an effect (Figure 3C one-way ANOVA F_2,24_ = 6.903 followed by Tukey’s multiple comparison test *p* < 0.01 SHAM vs. Aβ, and *p* < 0.05 Aβ vs. Aβ+AKBA). We can exclude that depressive-like behaviors tested could be related to alteration in locomotion since no difference was retrieved in the open field test quantifications (Supplementary Material, Figure S2).

### 3.3. Effect of Sublingual AKBA on Noradrenaline and Glutamate Content

**Figure 4:**
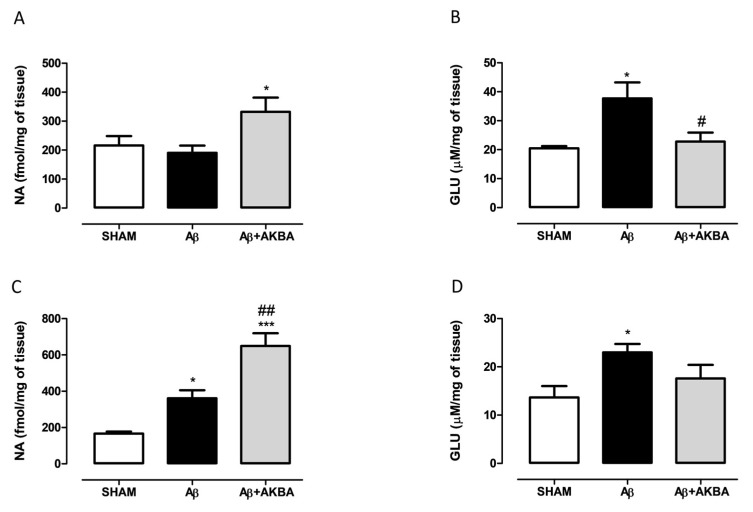
Effects of AKBA administration on NA and GLU content in PFC and HIPP of Aβ-treated mice. (**A**) NA (fmol/mg of tissue, SHAM n = 8, Aβ n = 6, Aβ+AKBA n = 6) and (**B**) GLU (uM/mg of tissue, SHAM n = 5, Aβ n = 5, Aβ+AKBA n = 6) levels in the PFC of mice 7 days after icv injection of vehicle (SHAM, 5 µL, white bar), Aβ (Aβ, 4 µM, black bar), and Aβ+AKBA (Aβ, 4 µM + sublingual AKBA, 5 mg/kg, gray bar). (**C**) NA ( fmol/mg of tissue, SHAM n = 6, Aβ n = 6, Aβ+AKBA n = 6) and (**D**) GLU (uM/mg of tissue, SHAM n = 6, Aβ n = 6, Aβ+AKBA n = 5) levels in the HIPP of mice 7 days after icv injection of vehicle (SHAM, 5 µL, white bar), Aβ (Aβ, 4 µM, black bar), and Aβ+AKBA (Aβ, 4 µM + sublingual AKBA, 5 mg/kg, gray bar). One-way ANOVA followed by Tukey’s multiple comparison test, * *p* < 0.05 Aβ versus SHAM; * *p* < 0.05, *** *p* < 0.001 Aβ+AKBA versus SHAM; # *p* < 0.05, ## *p* < 0.01 Aβ+AKBA versus Aβ.

In order to corroborate behavioural data with neurochemical quantification, NA and GLU levels were measured in PFC and HIPP of treated mice. As shown in Figure 4A, AKBA (given 30 min earlier) administration significantly increased NA levels in Aβ-treated mice (Figure 4A one-way ANOVA F_2,17_ = 3.986 followed by Tukey’s multiple comparison test *p* < 0.05 Aβ vs. Aβ+AKBA). Cortical GLU content was quantified, as evidenced by one-way ANOVA, and icv Aβ increased GLU levels compared to SHAM mice, and AKBA administration restored those levels to control (Figure 4B one-way ANOVA F_2,13_ = 6.493 followed by Tukey’s multiple comparison test *p* < 0.05 SHAM vs. Aβ, and *p* < 0.05 Aβ vs. Aβ+AKBA). Regarding the NA quantification, we found that Aβ induced a significant increase in catecholamine levels, and AKBA was able to evoke a further increase compared to SHAM mice (Figure 4C one-way ANOVA F_2,15_ = 25.28 followed by Tukey’s multiple comparison test *p* < 0.05 SHAM vs Aβ, and *p* < 0.01 Aβ vs. Aβ+AKBA, and *p* < 0.001 SHAM vs. Aβ+AKBA). In parallel, GLU levels were quantified in the same area, as revealed by statistical analyses; the peptide injection significantly increased hippocampal GLU levels, and AKBA prevented such an event (Figure 4D one-way ANOVA F_2,14_ = 4.429 followed by Tukey’s multiple comparison test *p* < 0.05 SHAM vs. Aβ, and *p* > 0.05 Aβ vs. Aβ+AKBA).

### 3.4. Effect of Sublingual AKBA on Serotonin and Kynurenine Content

**Figure 5:**
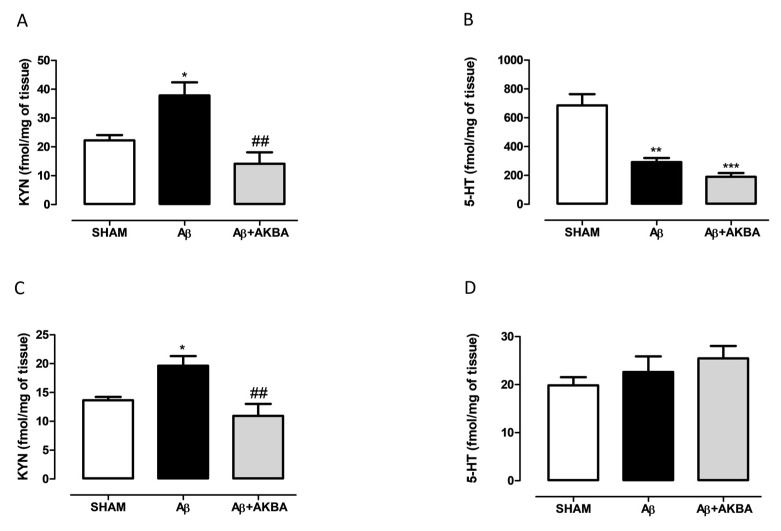
Effects of AKBA administration on KYN and 5-HT content in PFC and HIPP of Aβ-treated mice. (**A**) KYN (fmol/mg of tissue, SHAM n = 4, Aβ n = 5, Aβ+AKBA n = 5) and (**B**) 5-HT (fmol/mg of tissue, SHAM n = 7, Aβ n = 4, Aβ+AKBA n = 6) levels in the PFC of mice 7 days after icv injection of vehicle (SHAM, 5 µL, white bar), Aβ (Aβ, 4 µM, black bar), and Aβ+AKBA (Aβ, 4 µM + sublingual AKBA, 5 mg/kg, gray bar). (**C**) KYN (fmol/mg of tissue, SHAM n = 6, Aβ n = 6, Aβ+AKBA n = 5) and (**D**) 5-HT (fmol/mg of tissue, SHAM n = 6, Aβ n = 6, Aβ+AKBA n = 6) levels in the HIPP of mice 7 days after icv injection of vehicle (SHAM, 5 µL, white bar), Aβ (Aβ, 4 µM, black bar), and Aβ+AKBA (Aβ, 4 µM + sublingual AKBA, 5 mg/kg, gray bar). One-way ANOVA followed by Tukey’s multiple comparison test, * *p* < 0.0, ** *p* < 0.01 Aβ versus SHAM; *** *p* < 0.001 Aβ+AKBA versus SHAM; ## *p* < 0.01 Aβ+AKBA versus Aβ.

In order to understand the role played by the serotonergic system on AKBA effects, we quantified 5-HT and KYN levels in the PFC and HIPP of treated mice. As shown in Figure 5A, treatment induced a significant increase in cortical KYN levels compared to SHAM mice, while AKBA administration prevented such an increase (one-way ANOVA F_2,11_ = 10.32 followed by Tukey’s multiple comparison test *p* < 0.05 SHAM vs. Aβ and *p* < 0.01 Aβ vs. Aβ+AKBA). Regarding 5-HT levels, the peptide induced a significant decrease in PFC compared to SHAM mice, however no effect was retrieved after AKBA administration (Figure 5B one-way ANOVA F_2,14_ = 21.58 followed by Tukey’s multiple comparison test *p* < 0.05 SHAM vs. Aβ, and *p* < 0.001 SHAM vs. Aβ+AKBA). In the HIPP area, KYN levels followed the same pattern observed in the PFC. Indeed, AKBA administration prevented the increase in KYN levels induced by the peptide central administration (Figure 5C one-way ANOVA F_2,14_ = 8.673 followed by Tukey’s multiple comparison test *p* < 0.05 SHAM vs. Aβ and *p* < 0.01 Aβ vs. Aβ+AKBA). Conversely, no differences among experimental groups were found in the hippocampal 5-HT content (Figure 5D, one-way ANOVA F_2,15_ = 10.32 followed by Tukey’s multiple comparison test n.s.).

### 3.5. Effect of Sublingual AKBA on GFAP, CD11b and NFkB Levels

**Figure 6:**
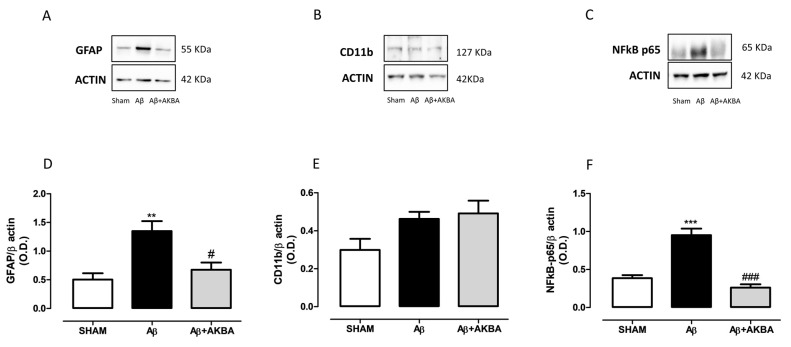
Effects of AKBA administration on cortical GFAP, CD11b and NFkB-p65 levels. Representative image of Western blotting of (**A**) GFAP, (**B**) CD11b and (**C**) NFkB-p65. (**D**) Quantification of the optical density of GFAP bands normalized to the actin protein value in the PFC of mice 7 days after icv injection of vehicle (SHAM, 5 µL, white bar), Aβ (Aβ, 4 µM, black bar), and Aβ+AKBA (Aβ, 4 µM + sublingual AKBA, 5 mg/kg, gray bar), (SHAM n = 5, Aβ n = 4, Aβ+AKBA n = 4). (**E**) Quantification of the optical density of CD11b bands normalized to the actin protein value in the PFC of mice 7 days after icv injection of vehicle (SHAM, 5 µL, white bar), Aβ (Aβ, 4 µM, black bar), and Aβ+AKBA (Aβ, 4 µM + sublingual AKBA, 5 mg/kg, gray bar), (SHAM n = 4, Aβ n = 4, Aβ+AKBA n = 4). Quantification of the optical density of NFkB-p65 bands normalized to the actin protein value in the PFC of mice 7 days after icv injection of vehicle (SHAM, 5 µL, white bar), Aβ (Aβ, 4 µM, black bar), and Aβ+AKBA (Aβ, 4 µM + sublingual AKBA, 5 mg/kg, gray bar), (**F**) SHAM n = 4, Aβ n = 4, Aβ+AKBA n = 4). One-way ANOVA followed by Tukey’s multiple comparison test, ** *p* < 0.01, *** *p* < 0.001 Aβ versus SHAM; # *p* < 0.05, ### *p* < 0.001 Aβ+AKBA versus Aβ.

Behavioural and neurochemical data were further corroborated by biochemical quantifications. In particular, we tested GFAP and Cd11b along with NF-kB in both PFC and HIPP. Figure 6A,D showed a significant increase in GFAP levels in the PFC of Aβ-injected animals compared to SHAM mice, while AKBA administration prevented such an increase (Figure 6A,D, one-way ANOVA F_2,10_ = 10.68 followed by Tukey’s multiple comparison test *p* < 0.01 SHAM vs. Aβ and *p* < 0.05 Aβ vs. Aβ+AKBA). Concerning cortical CD11b content, no differences were found among experimental groups (Figure 6B,E, one-way ANOVA F_2,9_ = 3.472 followed by Tukey’s multiple comparison test n.s.). As shown in Figure 6C,F, the Aβ peptide administration significantly increased cortical NF-kB levels, while AKBA treatment prevented such an increase (Figure 6C,F, one-way ANOVA F_2,9_ = 37.25 followed by Tukey’s multiple comparison test *p* < 0.001 SHAM vs. Aβ and Aβ vs. Aβ+AKBA).

**Figure 7:**
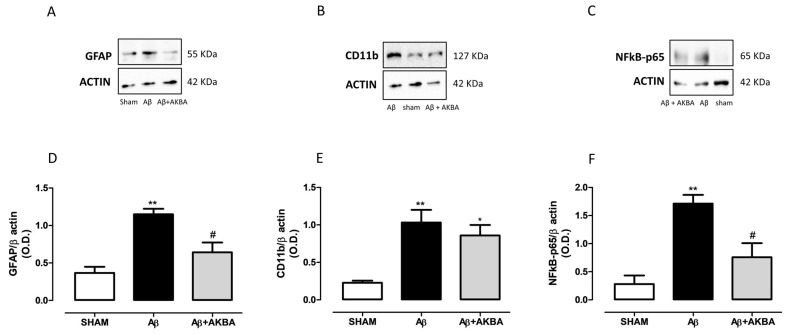
Effects of AKBA administration on hippocampal GFAP, CD11b and NFkB-p65 levels. Representative image of Western blotting of (**A**) GFAP, (**B**) CD11b and (**C**) NFkB-p65. (**D**) Quantification of the optical density of GFAP bands normalized to the actin protein value in the HIPP of mice 7 days after icv injection of vehicle (SHAM, 5 µL, white bar), Aβ (Aβ, 4 µM, black bar), and Aβ+AKBA (Aβ, 4 µM + sublingual AKBA, 5 mg/kg, gray bar), (SHAM n = 5, Aβ n = 3, Aβ+AKBA n = 5). (**E**) Quantification of the optical density of CD11b bands normalized to the actin protein value in the PFC of mice 7 days after icv injection of vehicle (SHAM, 5 µL, white bar), Aβ (Aβ, 4 µM, black bar), and Aβ+AKBA (Aβ, 4 µM + sublingual AKBA, 5 mg/kg, gray bar), (SHAM n = 4, Aβ n = 5, Aβ+AKBA n = 4). (**E**) Quantification of the optical density of NFkB-p65 bands normalized to the actin protein value in the PFC of mice 7 days after icv injection of vehicle (SHAM, 5 µL, white bar), Aβ (Aβ, 4 µM, black bar), and Aβ+AKBA (Aβ, 4 µM + sublingual AKBA, 5 mg/kg, gray bar), (**F**) SHAM n = 3, Aβ n = 4, Aβ+AKBA n = 4). One-way ANOVA followed by Tukey’s multiple comparison test, ** *p* < 0.01 Aβ versus SHAM; * *p* < 0.05 Aβ+AKBA versus SHAM; # *p* < 0.05 Aβ+AKBA versus Aβ.

As shown in Figure 7A,D, the Aβ injection induced a significant increase in hippocampal GFAP levels compared to SHAM mice, whereas AKBA administration restored those levels back to control (Figure 7A,D, one-way ANOVA F_2,10_ = 11.31 followed by Tukey’s multiple comparison test *p* < 0.01 SHAM vs. Aβ and *p* < 0.05 Aβ vs. Aβ+AKBA). When CD11b levels were quantified, significant enhanced hippocampal levels were retrieved after Aβ icv administration, however AKBA was not able to modify such an outcome (Figure 7B,E, one-way ANOVA F_2,10_ = 9.385 followed by Tukey’s multiple comparison test *p* < 0.01 SHAM vs. Aβ and *p* < 0.05 SHAM vs. Aβ+AKBA). Ultimately, we determined NF-kB content in the same area. As shown in Figure 7C,F, the administration of the Aβ peptide significantly increased the levels of this transcription factor while AKBA treatment prevented such an increase (Figure 7C,F, one-way ANOVA F_2,8_ = 12.83 followed by Tukey’s multiple comparison test *p* < 0.01 SHAM vs. Aβ and *p* < 0.05 Aβ vs. Aβ+AKBA).

## 4. Discussion

In the present study, we showed the protective effect of sublingual administration of AKBA on the depressive-like profile induced by the icv injection of Aβ in mice. In particular, we first decided to verify the effective capacity of AKBA to cross the blood brain barrier and to reach effectively cerebral areas. Indeed, this compound, along with other boswellic acids, is characterized by low oral bioavailability [24]. Accordingly, in vitro study in Caco-2 cells, a well-accepted model to simulate human intestinal absorption, revealed only moderate to poor permeability for boswellic acids and low permeability for AKBA [32]. This outcome can be explained on the basis of their high hydrophobicity and in turn very low water solubility, justifying the negligible quantity orally absorbed [33]. Therefore, such poor bioavailability is a major limitation to the efficacy of this herbal product. Thus, improving the bioavailability of Boswellia active ingredients is very advantageous to better profit from the therapeutic potential of this food supplement.

In this light, we tested the sublingual administration route since sublingual food supplements are growing in popularity and they are more often studied in clinical trials [34]. Indeed, the sublingual route is considered an attractive alternative for the consumption of food characterized by low bioavailability, considering that after sublingual administration, substances are rapidly absorbed and then distributed throughout the body by-passing the liver first-pass metabolism or possible modifications due to the crossing of the digestive tract. In our experimental conditions, we compared the sublingual route of administration with another route of administration equally characterized by avoiding the liver first-pass metabolism—the intranasal route of administration. Comparing AUCs for cerebral AKBA concentrations after these types of administrations, we found that at the same dose, the compound rapidly crossed the blood brain barrier, but the sublingual route of administration was characterized by higher bioavailability for AKBA. However, we cannot completely rule out that the different dissolving conditions might have affected the brain penetrance of AKBA. In this regard, we could not use a sunflower vehicle for intranasal administration because of its viscosity; further studies will be undertaken for dispelling any doubts. At any rate, our data indicate that sublingual administration of AKBA appears to be a suitable route of administration of this compound. Furthermore, by pursuing this therapeutic strategy we can afford to use a lower dosage of this compound, counteracting possible emerging side effects. Accordingly, no gross signs of toxicity were retrieved in the present study. In particular, no alteration in general locomotion was assured, as well as other signs of toxicity, such as sedation/excitation, piloerection, alterations of respiration rate, and tremors and stereotypies, allowing a complete benefit of AKBA positive effects. Indeed, AKBA holds antioxidant and anti-inflammatory properties, and it is considered a 5-LOX inhibitor. The role of this compound in aging and inflammation is emerging, thus in the present study, we evaluated the effects of AKBA in a mouse model of Aβ-induced toxicity. In the present work, we reproduced in mice the rat model of Aβ-induced depressive-like phenotype. Here, we performed two tests widely used in mice to evaluate different aspects of depressive-like behavior, such as the ST and the TST.

We found that Aβ induced a loss of self-care and motivational behavior in the ST, considering that, compared with SHAM, Aβ-treated mice demonstrated a decrease in time spent in self-care and increased latency time to leak the sweet/viscous solution sprayed on their back. Likewise, Aβ increased the time spent in performing immobility in the TST. Indeed, the lack of escape-related behavior is considered a behavior reminiscent of depressive-like symptoms in mice. This test has been validated for the assessment of antidepressant efficacy of drugs, but it is also used to evaluate the outcomes after environmental or neurobiological manipulation leading to a depressive state [35,36].

In our experimental conditions, we showed that sublingual AKBA administration was able to counteract both behavioral alterations induced by Aβ administration, indicating that this compound holds both anti-anhedonia- and antidepressant-like properties. Interestingly, although the highest cerebral concentration of AKBA occurred 5 min after its administration, our data indicated that beneficial effects were not occurring at those earlier time points, but at a later time instead. Thus, according to behavioral outcomes, neurochemical and biochemical quantifications were carried out at 30 minute time points, supporting the hypothesis that the mechanisms of action of AKBA related to its anti-depressant effects are not directly linked to AKBA presence in the brain, but to consequent action occurring in a longer period. In trying to understand a possible mechanism explaining the beneficial effect of AKBA, neurochemical quantifications were carried out. In the present paper, we chose two different areas known to be involved in depression, and known to be sensitive to Aβ toxicity also in line with our previous experience in rats [9,37], i.e., PFC and HIPP. According to our previous experience, here we found that these areas were vulnerable to Aβ peptide effects. Regarding the glutamatergic system, here we found that Aβ icv administration caused a significant increase in GLU levels in both the PFC and HIPP. It has been postulated that alteration in glutamate transport, along with reduced conversion into glutamine, can prompt to enhance concentrations of GLU at the synaptic level, then priming excitotoxicity ending up in cell death after an increased release of intracellular Ca2+ concentrations secondary to NMDA receptor activation [38,39]. Our data are in line with literature previously published, considering that up-regulation of glutamatergic presynaptic buttons has been reported both in mild cognitive impairment and in mouse models of AD [40,41]. It is worth noting that the involvement of the glutamatergic system has also been reported for inducing a depressive state [42] and the blocking of NMDA receptors has often been reported as a novel pharmacological strategy to treat depressive disorders [43]. In this regard, we have previously found that acute ketamine treatment is able to revert the pro-depressive-like effect of Aβ in rats [43]. In our model, we found that the antidepressive effect of AKBA administration was accompanied by reduced GLU overproduction secondary to Aβ injection. It has been previously shown that AKBA has a synergistic effect with COX-2 inhibitors in counteracting excitotoxic damage in vivo [44] and an anti-glutamatergic property has been described for this compound when tested in a mouse model of AD [2].

In addition, in our model we found that this increase in GLU levels was paralleled by the enhancing of KYN contents. Interestingly, a crucial crosstalk between the KYN pathway and glutamatergic function has been described [20]. In particular, KYN derivates, such as quinolinic acid or kynurenic acid have been shown to oppositely interact with either ionotropic or metabotropic glutamate receptors, or vesicular glutamate transport [20]. In addition, as an indirect mechanism, it has been proposed that KYN metabolites can modulate glutamatergic functions differently through the modulation of oxidative stress-mediated effect [45,46]. Furthermore, it has been shown that AKBA can attenuate oxidative stress-mediated toxic effects of GLU [47], thus we can hypothesize that AKBA might be able to ameliorate Aβ-induced toxicity also through its antioxidant properties.

On the other hand, concerning the effect of Aβ on monoamines, AKBA showed a different mechanism of action in these two brain areas in mice. In particular, we found that the Aβ injection caused a significant reduction in 5-HT content in the PFC, while no alterations were evidenced in the HIPP. These data are in line with our previous observations that revealed in the same model applied to rats the serotonergic system is highly vulnerable to Aβ effect in the cortical area, while the hippocampal serotonergic system is not sensitive to the peptide injection [8,10,48,49]. Conversely, regarding the noradrenergic system, only the hippocampal area responded to the icv administration of the peptide. According to our previous observations, such a phenomenon is rapidly occurring in rats after Aβ injection as soon as two hours after the release of the peptide [27]. We have interpreted this outcome as an early compensatory effect rapidly occurring in response to the peptide injection lasting until 7 days after the injection endpoint. In our experience, such an increase is secondary to the inflammatory response and it is mediated by IL-1β or iNOS activation [27]. However, in rats this event appears in either the PFC or HIPP, while here we showed a species-specific response, considering that in mice such an increase was evidenced in the HIPP only. Interestingly, AKBA increased NA content in both areas. These outcomes, apparently surprising, are very similar to the neurochemical mechanisms underlying the antidepressant activity of ketamine [50]. Interestingly, a classical antidepressant, such as fluoxetine, also reduced the depressive-like behavior induced by Aβ through the increased levels of NA. In this regard, it has been reported that glial activation can be regulated by NA [51]. In addition, in vitro and our in vivo studies have highlighted the NA protective role against toxicity induced by Aβ, via the enhancement of neurotrophic factor expression through the β adrenergic receptor pathway [52,53].

In our experimental conditions, we found that the Aβ-induced toxicity was occurring via activation of glial cells, particularly microglia and astrocytes. In this regard, microglial activation secondary to Aβ exposure has been shown to enhance GLU release through GLU transport [54], and a vicious cycle has been evidenced with KYN interactions [55]. Likewise, astrocytes were also activated after Aβ injection in mice. It is well known that astrocytes play a crucial role in restoring brain homeostasis responding to brain insults by promoting an inflammatory and immune response [56]. In this way, a prominent role in astrocytes is played by the NF-kB pathway [56]. Interestingly, a pro-inflammatory role has been ascribed to astrocytic NF-κB. Indeed, disruption of glutamatergic re-uptake has been evidenced after the inhibition of astrocyte NF-κB [57]. In our conditions, AKBA was able only to reduce the expression of astrocytes, such as GFAP, but not microglial biomarkers, such as CD11b, indicating that the reduction in GLU and KYN content evoked by AKBA administration may be restricted at the astrocytic level. AKBA has been shown to negatively modulate NF-kB signaling. Accordingly, in our conditions, we found a significant reduction of NF-κB p65 expression after AKBA administration. Strategies targeting NF-κB p65 may prove to be very useful in the treatment of chronic inflammation-associated conditions, such as depression or early phase AD. Furthermore, the NF-kB pathway in astrocyte has been reported to mediate up-regulation of apolipoprotein E gene (APOE) [58] and COX-2 after Aβ exposure [59]. Both targets are crucially involved in the early and late phase of AD, thus AKBA as a potential therapeutic strategy might be very interesting. Nonetheless, AKBA can act as a 5-LOX inhibitor [60]. Inhibition of 5-LOX has been associated with reduced astrocytic activation and death [61]. Interestingly, in AD patients, human post-mortem evaluations reported up-regulation of 5-LOX expression [62]. In addition, genetic AD models revealed that 5-LOX overexpression worsened amyloidogenic production and Aβ-associated toxicity [63,64]. In this context, cysteinyl leukotrienes, downstream derivatives of 5-LOX activation, are potent lipid mediators involved in inflammation and in neurodegeneration [61] and significant increase in their receptor expression in the HIPP and PFC has been reported after Aβ injection [65]. Interestingly, 5-LOX, by interacting with the glutamatergic system, has been considered another potential target for depression treatment [66]. Thus, by acting also as a 5-LOX inhibitor, AKBA can indirectly modulate glutamatergic function through this mechanism.

## 5. Conclusions

Taken together, our data support the hypothesis that AKBA can be considered as a food supplement suitable as an adjuvant for therapy of depression in early-stage AD. Indeed, our results suggest that by reducing astrocytes activation and by reducing NF-kB activation, this compound is able to revert an in vivo Aβ-mediated increase in GLU and KYN and associated depressive-like phenotype.

Therefore, AKBA represents a possible strategy that by targeting GFAP/NF-κB and 5-LOX/NF-κB pathways can be considered as a valuable tool for counteracting Aβ-induced toxicity. Furthermore, the sublingual administration represents a valuable and appealing route for this food supplement consumption.

## Data Availability

The data presented in this study are available on request from the corresponding author.

## Supplementary Materials

The following are available online at https://www.mdpi.com/2218-273X/11/5/686/s1, Figure S1: Behavioural effects after 5 min from sublingual AKBA administration in Aβ-treated mice by using the splash test; Figure S2: Open field test (crossing time, seconds-sec) after 30 min of AKBA (n = 8) or vehicle (n = 8) administration. Student’s *t*-test, *p* > 0.05.
