# Supplementary material for: Sublingual AKBA Exerts Antidepressant Effects in the Aβ-Treated Mouse Model
Source: Biomolecules. 2021 May 3;11(5):686. doi: 10.3390/biom11050686 (PMC8170916; doi:10.3390/biom11050686)
Supplement: Supplementary file 1 [file biomolecules-11-00686-s001.zip › biomolecules-1184461-supplementary.pdf]

Supplementary Material

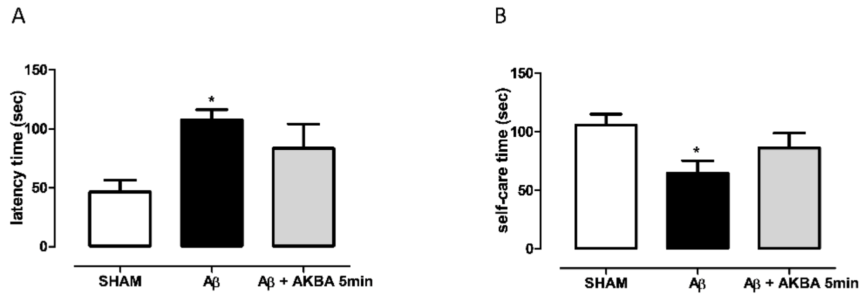

Figure S 1 Behavioural effects after 5 minutes from sublingual AKBA administration in Aβ-treated mice by using the splash test (A) Latency to leak (sec) in the splash test of mice 7 days after icv injection of vehicle (SHAM, 5µL, white bar), Aβ (Aβ, 4µM, black bar), and Aβ+AKBA 5min (Aβ, 4µM + sublingual AKBA, 5mg/kg, grey bar), (SHAM n=9, Aβ n=9, Aβ+AKBA 5min n=10), One-Way ANOVA followed by Tukey's post hoc test, $F_{(2,25)}=4.170$, *P<0.05 Aβ vs SHAM. (B) Time spent performing self-care (sec) in the splash test of mice 7 days after icv injection of vehicle (SHAM, 5µL, white bar), Aβ (Aβ, 4µM, black bar), and Aβ+AKBA 5min (Aβ, 4µM + sublingual AKBA, 5mg/kg, grey bar), (SHAM n=9, Aβ n=9, Aβ+AKBA 5min n=10). One-Way ANOVA followed by Tukey's post hoc test, $F_{(2,25)}=3.188$, *P<0.05 Aβ vs SHAM

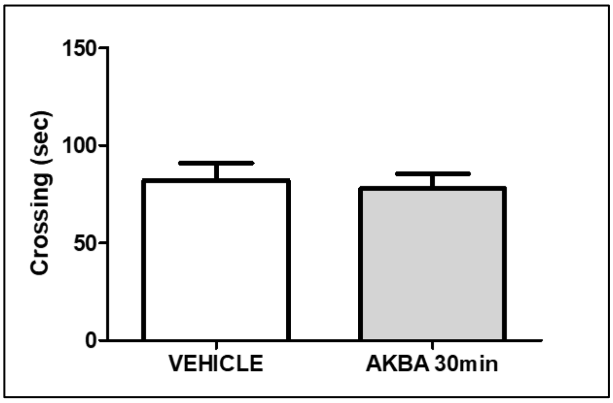

Figure S 2 Open field test (crossing time, seconds-sec) after 30 min of AKBA (n=8) or vehicle (n=8) administration. Student's *t*-test, P>0.05
